# Laxative activities of *Mareya micrantha *(Benth.) Müll. Arg. (Euphorbiaceae) leaf aqueous extract in rats

**DOI:** 10.1186/1472-6882-10-7

**Published:** 2010-02-16

**Authors:** Souleymane Méité, Calixte Bahi, Dodéhé Yéo, Jacques Y Datté, Joseph A Djaman, David J N'guessan

**Affiliations:** 1 Laboratoire de Pharmacodynamie Biochimique, UFR Biosciences, Université de Cocody-Abidjan, 22 BP 582 Abidjan 22, Côte d'Ivoire; 2 Laboratoire de Nutrition et Pharmacologie, UFR Biosciences, Université de Cocody-Abidjan, 22 BP 582 Abidjan 22, Côte d'Ivoire

## Abstract

**Background:**

*Mareya micrantha *(Benth.) Müll. Arg. (Euphorbiaceae) is a shrub that is commonly used in Côte d'Ivoire (West Africa) for the treatment of constipation and as an ocytocic drug. The present study was carried out to investigate the laxative activity of *Mareya micrantha *in albino's Wistar rats.

**Methods:**

Rats were divided in 5 groups of 5 animals each, first group as control, second group served as standard (sodium picosulfate) while group 3, 4 and 5 were treated with leaf aqueous extract of *Mareya micrantha *at doses of 100, 200 and 400 mg/kg body weight (b.w.), *per os *respectively. The laxative activity was determined based on the weight of the faeces matter. The effects of the leaves aqueous extract of *Mareya micrantha *and castor oil were also evaluated on intestinal transit, intestinal fluid accumulation and ions secretion.

**Results:**

Phytochemicals screening of the extract revealed the presence of flavonoids, alkaloids, tannins, polyphenols, sterols and polyterpenes. The aqueous extract of *Mareya micrantha *applied orally (100, 200 and 400 mg/kg; *p.o*.), produced significant laxative activity and reduced loperamide induced constipation in dose dependant manner. The effect of the extract at 200 and 400 mg/kg (*p.o*.) was similar to that of reference drug sodium picosulfate (5 mg/kg, *p.o*). The same doses of the extract (200 and 400 mg/kg, *p.o*.) produced a significant increase (p < 0.01) of intestinal transit in comparison with castor oil (2 mL) (p < 0.01). Moreover, the extract induced a significant enteropooling and excretion of Cl^-^, Na^+^, K^+ ^and Ca^2+ ^in the intestinal fluid (p < 0.01).

**Conclusions:**

The results showed that the aqueous extract of *Mareya micrantha *has a significant laxative activity and supports its traditional use in herbal medicine.

## Background

Constipation is a highly prevalent, often chronic gastrointestinal disorder that affects adults [1-3]. The treatment with classic drugs did not cut, in one hand with the inadequate relief of bloating and other symptoms, and with the lack of efficacy in relieving constipation. So far, half of patients were not satisfied with the effect of laxatives on improving quality of life [4,5].

Plants have long been a very important source of drugs against several diseases including constipation. *Mareya micrantha *is well known in the traditional medical practice of the south of Côte d'Ivoire (West Africa) where the leaves of this plant is extracted with water and the extract is taken orally for the treatment of constipation [6]. It is commonly called "oyia" in the local language of "Attié" [6]. *Mareya micrantha *(Benth.), Syn. *Mareya spicata *(Baill), Müll. Arg. (Euphorbiaceae) is a shrub that is found in west and central Africa. The plant flourishes in tropical climate and it is commonly used in different parts of west and central Africa to treat diseases which require drastic action, such as tapeworm infections, gonorrhea and leprosy [7].

Previous studies showed that the aqueous leaf extracts of *M. micrantha *suppressed cardiac contractility of isolated frog and rat hearts in a concentration dependent way [8,9]. In another test, an aqueous leaf extract elicited concentration-dependent contractions of the longitudinal muscle of isolated guinea-pig ileum [10]. Leaf extracts caused hypotension in dogs, and a root extract caused paralysis of the respiratory centre in rats. The methanol and cold aqueous extracts of the leaves showed antibacterial activity against *Enterobacter aerogenes*, *Agrobacterium tumefaciens*, *Bacillus subtilis*, *Clostridium sporogenes*, *Escherichia coli *and *Staphylococcus aureus *[7]. Ethanolic leaf extracts showed low antiplasmodial activity against a chloroquine-resistant strain of *Plasmodium falciparum *[11].

Although quite a number of scientific investigations have been undertaken to validate the local use of this plant, there seems to be no report on the laxative activity of the leaves of the plant. The present study was planned to examine the laxative activity of the aqueous extract of *Mareya micrantha *leaves.

## Methods

### Plant material

The *Mareya micrantha *leaves, used for the study were collected from Mareya *micrantha *plants located at Akoupé (south of Côte d'Ivoire, West Africa) in June 2007. The plant was identified and authenticated by Pr AKE ASSI at the Department of Botany, University of Cocody. After identification, a voucher specimen (N°18041) was deposited at the herbarium of "Centre National de Floristique" of the University of Cocody-Abidjan.

### Extraction procedure

The harvested leaves were air-dried and then reduced to powder with mortar and pestles. 80 g of the powder was extracted at a temperature of 90°C (by the process of maceration) with 2 L of distilled water. The macerated mixture was filtered and the filtrate was evaporated in a carefully regulated water bath (maintained at temperature of 90°C) to yield 4.5 g of dark solid extract. The extract was stored at a temperature of - 4°C pending the time for biological investigations.

### Animals

Albinos Wistar rats weighing 150-200 g were housed and bred in the animal house of UFR-Biosciences at Cocody University in Abidjan (Côte d'Ivoire). The animals were kept in standard cages with good ventilation, free access to feeds and water.

Experimental procedures and protocols used in this study were approved by ethical committee of Health Sciences, University of Cocody-Abidjan. These guidelines were in accordance with the internationally accepted principles for laboratory use and care.

### Phytochemical screening

The aqueous extract of *Mareya micrantha *was screened for phytochemical constituents using standard procedures of analysis [12].

### Gastrointestinal motility tests

The method of Mascolo *et al*. [13] was used. Rats were divided into different groups of five rats each and fasted for 18 hours before the experiment. Three of the groups were then treated orally with three increasing doses (100, 200 and 400 mg/kg) of the extracts serving as the test groups. One group served as blank or negative control treated with saline (5 mL/kg, *p.o*.) and the last group was administered castor oil (2 mL/rat, *p.o*.), a laxative agent, as the positive control. After 30 min, the animals were given 1 mL of freshly prepared charcoal meal (distilled water suspension containing 10% gum acacia, 10% vegetable charcoal). Following 30 min of charcoal administration, the rats were sacrificed by cervical dislocation and the abdomen immediately cut open, to excise the whole small intestine (pylorus region to caecum). The length of the small intestine and the distance between the pylorus region and the front of the charcoal meal was measured for obtaining the charcoal transport ration or percentage.

### Water and electrolyte secretion

The method of Robert *et al*. [14] was used. Animals of the first group received saline solution (5 mL/kg, *p.o*). Groups 2, 3, 4 and 5 received respectively castor oil (2 mL/rat, *p.o*) and increasing doses of the aqueous extract of *Mareya micrantha *(100, 200 and 400 mg/kg, *p.o*). Two hours later, the rats were sacrificed and the small intestine from the pylorus to caecum was extracted. The intestinal contents were collected by milking into a graduated tube and their volume was measured [15]. The fluid samples were analyzed for Na^+^, K^+^, Cl^- ^and Ca^2+ ^contrentrations using flame photometer (Elico^® ^CL361).

### Laxative activity

The method of Capasso *et al*. [16] was followed for this activity. Rats fasted for 12 h before the experiment were placed individually in cages lined with clean filter paper. Rats were divided in five groups with the first group acting as the control and administered saline (5 mL/kg, *p.o*.) that acted as the negative control. The second group received sodium picosulfate (5 mg/kg, *p.o*), this served as the positive control. The third, fourth and fifth groups received 100, 200 and 400 mg/kg *per os *of the *Mareya micrantha *aqueous extract. The faeces production (total number of normal as well as wet faeces) in all five groups was monitored for 16 h.

### Laxative activity on loperamide induced constipation

This study was carried out, as earlier described by Takahura *et al*. [17]. Rats were placed individually in cages lined with clean filter paper, allowed to fast for 18 hours and divided into five groups of five animals each. The aqueous extract of *Mareya micrantha *(100, 200, and 400 mg/kg, *p.o*.) was administered *per os *to the first three groups of rats. One of the two remaining other group received normal saline (5 mL/kg, *p.o*) and served as a control. The last group received *per os *the standard drug sodium picosulfate (5 mg/kg). After 1 h, all the animals received Loperamide (5 mg/kg, *p.o*.) by gavage. The faeces production (total number of normal as well as wet faeces) in all five groups was monitored for 8 h.

### Statistical analysis

Data obtained are presented as means ± standard error of mean (S.E.M.) for the number of animals in each group (n = 5). The differences between the data obtained from 'test' animal groups and the data obtained from untreated animal groups, were subjected to one-way analysis of variance (ANOVA; 95% confidence interval), followed by Dunnett's test. Values with p < 0.05 compared with the control group were considered as being significantly different.

## Results

### 1-Phytochemical screening

The phytochemical screening with the different tests described (see material and methods) revealed the presence of alkaloids, tannins, flavonoids, polyphenols, sterols and polyterpenes.

### 2-Effect of the aqueous extract of *M. micrantha* on gastrointestinal motility

The results of gastrointestinal motility test were reported in table 1. The aqueous extract of *Mareya micrantha *increased propulsion of the charcoal meal through the gastrointestinal tract in a concentration dependant manner. No significant effect was observed at the dose of 100 mg/kg of the aqueous extract of *Mareya micrantha*, but the doses of 200 mg/kg and 400 mg/kg (*p.o*.) of the extract produced a significant increase in the propulsion of charcoal meal compared to control group (normal saline, 5 mL/kg, *p.o*.) (p < 0.01). Castor oil (2 mL/rat, *p.o*.) produced greater gastrointestinal motility effect than the highest dose of the extract (400 mg/kg, *p.o*.) used.

**Table 1:** Effects of *M. micrantha *aqueous extract (MAR) on gastrointestinal motility in rats

Treatment	Dose	Percentage of distance (%)
Control	5 mL/kg	68,78 ± 4,64
Castor oil	2 mL/rat	93.71 ± 2.74***
MAR	100 mg/kg	70.54 ± 5.48
MAR	200 mg/kg	87.77 ± 1.84**
MAR	400 mg/kg	91.41 ± 2.11**

Values are expressed as mean ± S.E.M (n = 5); ** p < 0.01 compared to control group; and ***p < 0.001 compared to control group.

### 3-Effect on intestinal water secretion

The results of the volume of intestinal fluid analysis for control group, the extract at doses of 100, 200 and 400 mg/kg (*p.o*.) and castor oil (2 ml/rat, *p.o*.) are shown in table 2. Both doses of the extract (100 and 200 mg/kg) produced no significant effects on intestinal water secretion. However, the fluid volume of the rat intestine was significantly increased by the extract at the dose 400 mg/kg (*p.o*.) when compared with the untreated animals (control), which received only normal saline (p < 0.01). There was no statistical difference between castor oil (2 mL/rat, *p.o*.) and the extract at the dose of 400 mg/kg (*p.o*.).

**Table 2:** Effects of *M. micrantha aqueous *extract (MAR) on intestinal water secretion in rats

Treatment	Dose	Volume of intestinal fluid(mL)
Control	5 mL/kg	0.74 ± 0.15
Castor oil	2 mL/rat	3.06 ± 0.25**
MAR	100 mg/kg	1.54 ± 0.12
MAR	200 mg/kg	1.8 ± 0.70
MAR	400 mg/kg	2.92 ± 0.58**

Values are expressed as mean ± S.E.M (n = 5); ** p < 0.01 compared to control.

### 4-Effect on intestinal ion secretion

The results of intestinal ion secretion test were reported in table 3. There was no significant effect with the doses 100 and 200 mg/kg (*p.o*.) of the extract on intestinal Na^+^, K^+^, Cl^- ^secretion compared with control. At the dose of 400 mg/kg, the aqueous extract of *Mareya micrantha *increased signicantly Na^+^, K^+ ^(p < 0.05), Cl^- ^(p < 0.01) and Ca^2+ ^(p < 0.001) secretions through the gastrointestinal tract compared with control group. A similar effect in the gastrointestinal transit of charcoal meal in rats was obtained with castor oil (2 mL/rat, *p.o*.).

**Table 3:** Effects of *M. micrantha *aqueous extract on intestinal ion secretion in rats

Treatment	Dose	**Na**^+^**(mg/L)**	**K**^+^**(mg/L)**	**Cl**^-^**(mg/L)**	**Ca**^2+^**(mg/L)**
Control	5 mL/kg	2.160 ± 0.27	0.196 ± 0.01	13.96 ± 1.69	0.478 ± 0.07
Castor oil	2 mL/rat	3.99 ± 0.66*	0.380 ± 0.5*	29.81 ± 2.03***	0.782 ± 0.04**
MAR	100 mg/kg	2.288 ± 0.21	0.168 ± 0.02	15.68 ± 0.70	0.650 ± 0.06
MAR	200 mg/kg	2.566 ± 0.25	0.254 ± 0.03	19.86 ± 2.31	0.690 ± 0.02*
MAR	400 mg/kg	3.756 ± 0.50*	0.356 ± 0.06*	23.89 ± 2***	0.748 ± 0.04**

Values are expressed as mean ± S.E.M (n = 5); p < 0.05 ** compared to control group; **p < 0.01 compared to control group; and ***p < 0.001 compared to control group.

### 5-Laxative activity of aqueous extract of *M. micrantha*

In this study, the different doses of the extract showed dose dependant increase in fecal output of rats when compared to the control group (table 4). There was no significant difference between the extract at the dose of 100 mg/kg (*p.o*.) and control group. The effects of *Mareya micrantha *at doses of 200 and 400 mg/kg (*p.o*.) increased significantly fecal output of rats compared to control group (p < 0.05-0.01). The effect of the extract at the dose of 400 mg/kg (*p.o*.) was similar to that of the standard drug sodium picosulfate (5 mg/kg, *p.o*.).

**Table 4:** Laxative activity of aqueous extract of *M. micrantha *(MAR) in rats

Treatment	Dose	Faeces out put (g)	
		0-8 h	8 h-16 h
Control	5 mL/kg	0.748 ± 0.42	1.608 ± 0.65
Sodium picosulfate	5 mg/kg	5.090 ± 1.11**	5.415 ± 0.61**
MAR	100 mg/kg	0.892 ± 0.17	1.190 ± 0.28
MAR	200 mg/kg	3.703 ± 0.77*	4.733 ± 0.1.10*
MAR	400 mg/kg	4.829 ± 0.92**	5.217 ± 0.59**

Values are expressed as mean ± S.E.M (n = 5); * p < 0.05 compared to control group; and **p < 0.01 compared to control group.

### 6-Effect of the aqueous extract of *M. micrantha* on loperamide induced constipation in rats

In the loperamide-induced constipation, the aqueous extract of *Mareya micrantha *at the doses of 200 and 400 mg/kg (*p.o*.), increased the total number of faeces in a dose dependent manner, and the results were statistically significant (p < 0.05-0.01) (Table 5). There was no significant effect with the dose of 100 mg/kg (*p.o*.) of the extract compared with control. The reduction of the loperamide induced constipation at 400 mg/kg *(p.o.) *of plant extract treatment was found to be almost comparable with that of treatment by 5 mg/kg of sodium picosulfate.

**Table 5:** Effect of *M. micrantha *aqueous extract (MAR) on loperamide induced constipation in rat

Treatment	Dose	Weight of faeces (g)
Control	5 mL/kg	0.938 ± 0.45
Sodium picosulfate	5 mg/kg	3.84 ± 0.62**
MAR	100 mg/kg	2.602 ± 0.33
MAR	200 mg/kg	2.806 ± 0.42*
MAR	400 mg/kg	3.507 ± 0.45**

Values are expressed as mean ± S.E.M (n = 5); * p < 0.05 compared to control group; and

**p < 0.01 compared to control group.

## Discussion

The laxative activity of *Mareya micrantha *was studied in rats. The results showed that an oral administration of the leaves aqueous extract of *M. micrantha *produced significant and dose dependant increase in faeces output of rats in regards to the accumulation of water in intestinal loop and the stimulation of gastrointestinal motility. These effects were similar with that of castor oil (standard drug) at high dose of 400 mg/kg. Castor oil affects electrolyte transport and smooth muscle contractility in the intestine [18]. Its cathartic action is due to water accumulation in the intestine [19]. Castor oil causes diarrhea due to its active metabolite ricinoleic acid [20] which stimulates peristaltic activity in the small intestine, leading to changes in the electrolyte permeability of the intestinal mucosa. Its action also stimulates the release of endogenous prostaglandin [21]. The observed activities therefore suggest that laxative activity of the aqueous extract of *M. micrantha *may be mediated through this mechanism of action of ricinoleic acid.

In the other hand, our results have indicated that *M. micrantha *and sodium picosulfate exert respectively opposite effects with loperamide on the gastrointestinal function. It is well documented that loperamide abolishes experimental osmotic diarrhea by acting on intestinal motility, and consequently reducing the flow entering the colon [22,23]. Sodium picosulfate is a member of the polyphenolic group of stimulant laxatives. Following oral administration, it is converted in the colon to an active form through the action of bacterial enzymes [24]. As a result, its effects are directed principally in the colon, where it stimulates peristalsis and, in common with other laxatives, reduces water reabsorption leading to the softening of stools. These results suggest that the aqueous extract of *M. micrantha *contains secondary metabolites which could act by this way.

This study has also shown that *M. micrantha *had stimulated Na^+^, K^+ ^and Cl^- ^secretion. Most of the naturally laxative exert their effects on the colonic epithelium by stimulating Cl^- ^secretion and/or inhibiting Na^+ ^absorption, resulting in an accumulation of fluid and subsequent increased colonic motility [25].

The results showed that the aqueous extract of *M. micrantha *increased the propulsion of charcoal meal. This result is in concordance with the findings of Traoré *et al*.[26] who have shown that the leaves aqueous extract of *M. micrantha *caused stimulant effects on the spontaneous motility of isolated rabbit ileum. In the other hand, Tsaï *et al*. [10] have put in evidence the presence of cholinergic active ingredients in the aqueous extract of *M. micrantha *using the longitudinal muscle of isolated guinea-pig ileum. The propulsion of charcoal meal is probably due to the increasing of peristaltic movement in rat gastrointestinal tract resulting from the stimulation of cholinergic receptors by *M. micrantha*.

The intestinal transit is controlled by both neural and myogenic mechanisms [27]. An increase of the contractile activity of the smooth layers in general is responsible for acceleration of intestinal propulsion. Several mediators and neurotransmitters govern these motor patterns. Acethylcholine is the main excitatory neurotransmitter in the enteric nervous system [28]. Thus the presence of cholinomimetic constituents in the plant extract can explain the usefulness of *M. micrantha *in constipation pointed out by the ethnobotanical informations [7].

The Presence of phytoconstituents like terpenoids, sterols, flavonoids, phenolic compounds, tannins and alkaloids [29] have been previously found to be responsible for laxative activities in plants. Phytochemical screening of the extract of *M. micrantha *revealed the presence of alkaloids, tannins, flavonoids, polyphenols, sterols and polyterpenes. These constituents may be responsible for the laxative activity of *M. micrantha*.

## Conclusions

This study has shown that *M. micrantha *has laxative effects in addition to the various physiological effects earlier reported by other authors. The results of this study justify the use of the leaves of *M. micrantha *as laxative in traditional medicine. Further studies may be directed at characterizing the bioactive ingredients that are responsible for the observed activity in the plant.

## Competing interests

The authors declare that they have no competing interests.

## Authors' contributions

SM carried out the laboratory studies, helped in analysis of data and preparation of manuscript. CB collected the plant material, obtained a voucher specimen and made substantial contributions to data acquisition. DY helped in the animal experiments and statistical analysis. AJD has been involved in revising the manuscript. JDN and YJD have been involved in acquisition, analysis and interpretation of data, revising the manuscript for substantial intellectual content and final approval of manuscript for submission. All authors read and approved the final manuscript.

## Pre-publication history

The pre-publication history for this paper can be accessed here:

http://www.biomedcentral.com/1472-6882/10/7/prepub

## References

[B1] Muller-Lissner S The patholophysiology, diagnosis and treatment of constipation Dtsch Artztebl Int 2009 106 424 432 10.3238/arztebl.2009.0424 PMC2704368 19623313

[B2] Higgins PD Johanson JF Epidemiology of constipation in North America: a systematic review Am J Gastroenterol 2004 99 750 759 10.1111/j.1572-0241.2004.04114.x 15089911

[B3] Bosshard W Dreher R SChnegg JF Bula CJ The treatment of chronic constipation in elderly people: an update Drugs Aging 2004 21 911 930 10.2165/00002512-200421140-00002 15554750

[B4] Johanson JF Kralstein J Chronic constipation: a survey of the patient perspective Aliment Pharmacol Ther 2007 25 599 608 17305761 10.1111/j.1365-2036.2006.03238.x

[B5] Bengtsson M Ohlsson B Psychological well-being and symptoms in women with chronic constipation treated with sodium picosulphate Gastroenterol Nurs 2005 28 3 12 10.1097/00001610-200501000-00002 15738724

[B6] N'guessan K Contribution à l'étude ethnobotanique chez les Krobou de la souspréfecture d'Agboville (Côte d'Ivoire) Thèse de Doctorat 3ème cycle 1995 Université d'Abidjan-Cocody, Botanique et biologie végétale

[B7] Mac Foy CA Cline EI *In vitro *antibacterial activities of three plants used in traditional medicine in Sierra Leone J Ethnopharmacol 1990 28 323 327 10.1016/0378-8741(90)90083-6 2335960

[B8] Guede-guina F Tsai CS Smith MO Vangah MM Washington B Ochillo RF The use of isoleted functional heart to pharmacologically characterize active ingredient in the aqueous extracts of *Mareya micrantha* J Ethnopharmacol 1995 45 19 26 10.1016/0378-8741(94)01190-B 7739223

[B9] Abo KJ-C Aka KJ Ehile EE Guede Guina F Traore F Effets cholinergiques d'un extrait aqueux brut de Mareya micrantha (Euphorbiacée) sur la pression et d'activité cardiaque Revue Med Pharm Afr 2000 5 11 20

[B10] Tsai CS Guede Guina F Smith MO Vangah MM Ochillo RF Isolation of cholinergic active ingredients in aqueouse extracts of *Mareya micrantha *using the longitudinal muscle of isolated guinea-pig ileum as a pharmacological activity marker J Ethnopharmacol 1995 45 215 222 10.1016/0378-8741(94)01219-P 7623487

[B11] Zirihi GN Mambu L Guédé-Guina F Bodo B Grellier P *In vitro *antiplasmodial activity and cytotoxicity of 33 West African plants used for the treatment of malaria J Ethnopharmacol 2005 98 281 285 10.1016/j.jep.2005.01.004 15814260

[B12] Silva GL Lee I Kinghorn AD Cannell RJP Special problems with the extraction of plants Methods in Biotechnology (Natural product Isolation) 1998 New Jersey: Humana press 343 363 full_text

[B13] Mascolo N Izzo AA Avtore G Barboto F Capasso F Nitric oxide and castor oil induced diarrhea J Pharmacol Exp Therap 1994 268 291 295 8301570

[B14] Robert A Nezamis JE Lancaster C Hanchar AJ Klepper MS Enteropooling assay, a test for diarrhea produced by prostaglandins Prostaglandins 1976 11 809 828 10.1016/0090-6980(76)90189-1 935512

[B15] Pazhani GP Subramanian N Arunchalam G Hemalatha S Ravichandran V Antidiarrheal potencial of *Elephantopus Scaber Linn *leaf extract Ind drugs 2001 38 269 271

[B16] Capasso F Mascolo N Autore G Romano V Laxatives and the production of autacoids by rat colon J pharm Pharmacol 1986 38 627 629 2876085 10.1111/j.2042-7158.1986.tb03097.x

[B17] Takaharu S Fujie M Yuji L Kouji M Hideo K Laxative and Antidiarrheal activity of polycarbophil in mice and rats Jpn J Pharmacol 2002 89 133 141 10.1254/jjp.89.133 12120755

[B18] Izzo AA Mascolo N Viola P Capasso F Inhibitors of nitric oxide synthase enhance rat ileum contractions induced by ricinoleic acid *in vitro* Eur J Pharmacol 1993 243 87 90 10.1016/0014-2999(93)90172-E 7504631

[B19] Chitme HR Chandra R Kaushik S Studies on Antidiarrheal activity on *calotropis gigantean *in experimental animals J Pharm Pharmaceut Sci 2004 7 70 75 15144737

[B20] Ammon HV Thomas PJ Phillips SF Effect of the oleic acid and ricinoleic acid net jejunal water and electrolyte movement J Clin Invest 1974 53 374 379 10.1172/JCI107569 11344549 PMC301478

[B21] Galvez J Zavzuelo A Crespo ME Lorente MD Ocete MA Jimenez J Anti-diarrhoeic activity of *Euphorbia hirta *extract and isolation of an active flavonoid constituent Planta Med 1993 59 333 336 10.1055/s-2006-959694 8372151

[B22] Hughes S Higgs NB Turnberg LA Loperamide has antisecretory activity in the human jejunum *in vivo* Gut 1984 25 931 935 10.1136/gut.25.9.931 6590431 PMC1432486

[B23] Lawrence RS Carol ASA Stephen GM John SF Mechanism of the Antidiarrheal effect of loperamide Gastroenterology 1984 86 1475 1480 6714575

[B24] Kim DH Hyun SH Shim SB Kobashi K The rôle of intestinal bacteria in the transformation of sodium picosulphate Jpn J Pharmacol 1992 59 1 5 10.1254/jjp.59.1 1507649

[B25] Chatsri D Sutthasinee P Watchareewan T Nateetip K Barakol Extracted from *Cassia Siamea *stimulates chloride secretion in rat colon J pharmacol Exp Ther 2005 314 732 734 10.1124/jpet.105.084210 15870391

[B26] Traoré F Dosso M Guede-Guede F Aka KJ Effet myostimulant d'un extrait aqueux de *Mareya micrantha *(Euphorbiaceae) sur l'activité contractile intestinale de lapin Ethnopharmacologie 2004 33 45 59

[B27] Huizinga JD Ambrous K Der-Silaphet T Cooperation between neural and myogenic mechanisms in the control of distension-induced peristalsis in the mouse small intestine J Physiol 1998 506 843 56 10.1111/j.1469-7793.1998.843bv.x 9503342 PMC2230746

[B28] Waterman SA Costa M The role of enteric inhibitory motoneurons in peristalsis in the isolated guinea-pig small intestine J Physiol 1994 477 459 468 7932234 10.1113/jphysiol.1994.sp020207 PMC1155610

[B29] Longanga-Otshudi A Vercruysse A Foriers A Contribution to the ethnobotanical, phytochemical and pharmacological studies of traditionally used medicinal plants in the treatment of dysentery and diarrhea in Lomela area, Democratic Republic of Congo (DRC) J Ethnopharmacol 2000 71 411 423 10.1016/S0378-8741(00)00167-7 10940578

